# Cavitation intensifying bags improve ultrasonic advanced oxidation with Pd/Al_2_O_3_ catalyst

**DOI:** 10.1016/j.ultsonch.2020.105324

**Published:** 2020-09-07

**Authors:** Maria Pappaterra, Pengyu Xu, Walter van der Meer, Jimmy A. Faria, David Fernandez Rivas

**Affiliations:** a Mesoscale Chemical Systems Group, Faculty of Science and Technology, MESA+ Institute for Nanotechnology, and University of Twente, PO Box 217, 7500 AE Enschede, The Netherlands; b Delft University of Technology, Delft, The Netherlands; c Catalytic Processes and Materials Group, Faculty of Science and Technology, MESA+ Institute for Nanotechnology, University of Twente, PO Box 217, 7500 AE Enschede, The Netherlands; d Oasen Water Company, PO BOX 122, 2800 AC Gouda, The Netherlands; e Membranes Science and Technology, Faculty of Science and Technology, University of Twente, P.O. Box 217, 7500 AE Enschede, The Netherlands

**Keywords:** Bubbles, Cavitation, Process intensification, Bubble bag, Advanced oxidation

## Abstract

Advanced oxidation processes can potentially eliminate organic contaminants from industrial waste streams as well as persistent pharmaceutical components in drinking water. We explore for the first time the utilization of Cavitation Intensifying Bags (CIB) in combination with Pd/Al_2_O_3_ catalyst as possible advanced oxidation technology for wastewater streams, oxidizing terephthalic acid (TA) to 2-hydroxyterephthalic acid (HTA). The detailed characterization of this novel reaction system reveals that, during sonication, the presence of surface pits of the CIB improves the reproducibility and thus the control of the sonication process, when compared to oxidation in non-pitted bags. Detailed reaction kinetics shows that in the CIB reactor the reaction order to TA is zero, which is attributed to the large excess of TA in the system. The rate of HTA formation increased ten-fold from ~0.01 μM*min^−1^ during sonication in the CIB, to ~0.10 μM*min^−1^ for CIB in the presence of the Pd/Al_2_O_3_ catalyst. This enhancement was ascribed to a combination of improved mass transport, the creation of thermal gradients, and Pd/Al_2_O_3_ catalyst near the cavitating bubbles. Further analysis of the kinetics of HTA formation on Pd/Al_2_O_3_ indicated that initially the reaction underwent through an induction period of 20 min, where the HTA concentration was ~0.3 μM. After this, the reaction rate increased reaching HTA concentrations ~6 μM after 40 min. This behavior resembled that observed during oxidation of hydrocarbons on metal catalysts, where the slow rate formation of hydroperoxides on the metal surface is followed by rapid product formation upon reaching a critical concentration. Finally, a global analysis using the Intensification Factor (IF) reveals that CIB in combination with the Pd/Al_2_O_3_ catalyst is a desirable option for the oxidation of TA when considering increased oxidation rates and costs.

## Highlights

• Cavitation Intensification Bags with Pd/Al_2_O_3_ catalyst improve oxidation processes.
• Mass transport and thermal gradients improved near cavitating bubbles.
• Intensification Factor analysis and ultrasonic reaction kinetics are unveiled.

## Graphical abstract

**Figure:**
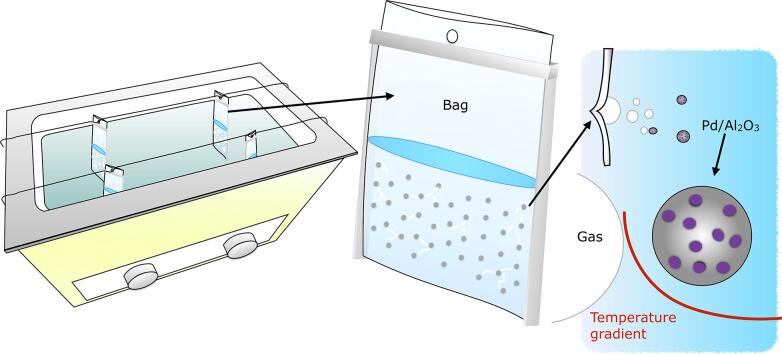

## 1 Introduction

Contamination of soil and groundwater from industrial waste streams poses serious health and environmental problems. Numerous innovative water treatment technologies have been proposed to tackle this issue, such as the Advanced Oxidation Processes (AOPs) [1], [2]. These promising processes are environmental-friendly methods based on the *in-situ* generation of powerful oxidizing agents, e.g. hydroxyl radicals (^.^OH), obtained at a sufficient concentration to effectively decontaminate the water [3]. Sonochemistry has been proposed as an AOP, which relies on ultrasound to induce cavitation in liquids [4], [5]. The use of ultrasonic waves has been considered as a simple, inexpensive, and valuable tool in chemistry because of its green character while promoting faster and selective transformations [5], [6]. In particular, sonochemistry is properly aligned with the first Principle of Green Engineering in ensuring that “all material, energy inputs and outputs are as inherently non-hazardous as possible”, because, in principle, no additives are needed to obtain positive results [7]. Hydroxyl radicals and other oxidative radical species have been applied in the treatment of industrial wastewater [8]. However, large scale application of several AOP, and particularly ultrasonication, are still very limited due to cost and inadequate information about the resultant water quality [8], [9], [10].

Sonochemical reactors have been identified as an effective tool to influence reaction mechanisms and its rate, due to the large amount of energy released, accompanied by the generation of free radicals, and enhanced mass transfer in multiphasic systems (gas–liquid and liquid–solid) [11]. Unfortunately, the low energy efficiency of most sonochemical systems, and the complex interdependence of several parameters leading to reproducibility issues have prevented a wider adoption by the industry [12], [13]. The use of microfluidics to control cavitation phenomena have led to increased efficiency of micro-sonoreactors [14]. However, the numbering up and scaling up of such microreactors to serve volumes relevant to industrial applications is still ongoing [15].

**Fig. 1:**
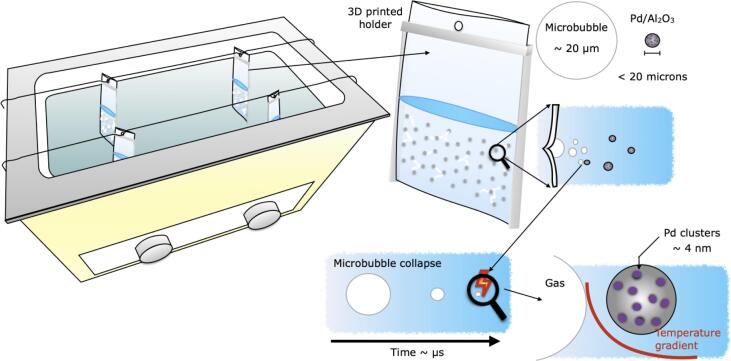
A schematic representation of the experimental setup and subcomponents. The CIBs have been numbered-up (to four) and placed on top of the active areas in the ultrasonic bath. Cavitation is enhanced by the presence of pits (indentations on the CIBs inner walls). Suspended catalyst particles next to the increased cavitation activity is the most salient feature of this study.

Cavitation Intensifying Bags (CIB) --commercialized as BuBble bags-- have been used for disparate applications, such as cleaning, emulsification and exfoliation [16], [17], [18], [19]. It is an example of Process intensification following the *Structure* approach [20], because of its working principle based on modified internal surfaces to entrap gas bubbles when filled with a liquid (see Fig. 1). CIBs are plastic bags with its inner walls modified to include pits (indentations). There are ca. 900 pits inside the CIBs, with a spacing of 3.5 mm. The pits have a diameter between 100 and 500 µm and a depth of 100–200 µm. The potential of CIB to help in the homogeneous distribution of the ultrasound energy inside a sonication bath, an essential feature to ensure reproducible and comparable results [21], has been demonstrated earlier. Upon exposure to ultrasound, the entrapped gas bubbles serve as nucleation sites of microbubbles generated in large. These CIBs are indeed able to enhance cavitation (radical formation and mechanical effects).

When heterogeneous catalysts are added to sonochemical reaction systems, rate enhancements of up to ten-fold can be achieved, preserving thermally sensitive reaction species that are inaccessible using conventional thermal-catalysis, where elevated temperatures and pressures are often required to achieve equivalent reaction rates [22], [23], [24]. The challenge is that controlling the acoustic induced and catalytic molecular transformations is often complex as the time- and length-scales at which these processes occur are orders of magnitude different [25], [26]. Thus, taming the cavitation process to control the catalysis can increase the energy efficiency and productivity of sonocatalytic processes. Here, we employed Pd catalyst supported on alumina (Pd/Al_2_O_3_) suspended inside CIBs to study the sonocatalytic oxidation terephthalic acid (TA) to 2-hydroxyterephthalic acid (HTA); a prototypic reaction employed for dosimetry of ionizing radiation. Understanding the behavior of CIB reactors for sonocatalytic oxidation is essential to assess its potential for low-temperature advanced oxidation processes for removing persistent pharmaceuticals in drinking water [27].

## 2 Experimental section

### 2.1 Materials

Commercial γ-Al_2_O_3_ powder purchased from BASF with a surface area of 195 m^2^/g, was used as catalyst support in this study. Tetra-ammine-palladium (II) nitrate solution (10 wt% in H_2_O, 99.99%), purchased in Sigma-Aldrich, was used as catalyst precursor. Aqueous ammonium solution (50% v/v water, Sigma-Aldrich) is used to adjust the pH for the catalyst preparation. All aqueous solutions were prepared using ultra purified water obtained from a water purification system (Millipore, Synergy).

### 2.2 Catalyst preparation

The preparation method is described in detail elsewhere [28]. In brief, Pd/γ-Al_2_O_3_ catalyst containing 5 wt% of Palladium, was prepared by wet impregnation. Typically, 10 g of the sieved support (particles smaller than 38 μm, mean particles size 22 µm) was calcined at 600 °C for 4 h to remove any organic contamination. Te support was suspended in 100 mL milliQ water and the pH of the solution was adjusted to 9 by adding 2 mL ammonia solution, as checked with a pH meter (Hanna instruments, pH 209). Then, 15 g of the palladium precursor solution (Pd(NO_3_)_2_·4NH_3_) was slowly added to the suspension. The suspension was stirred at room temperature for at least 1 h. The solution was transferred to a rotary evaporator to remove the liquid for 2 h. Finally, the catalyst was calcined in air at 400 °C for 3 h (heating rate 5 °C/min), followed by reduction in hydrogen (30 mL/min) diluted with nitrogen (total 60 mL/min) at the same temperature for 3 h.

### 2.3 Catalyst characterization

The Pd loading was determined with X-ray fluorescence spectroscopy, XRF (Philips PW 1480). CO chemisorption at room temperature was used to determine the accessible metal surface area (Chemisorb 2750, Micromeritics). Typically, the sample was reduced at room temperature in hydrogen for 1 h and then flushed with He at the same temperature for 0.5 h. Then CO was introduced as pulses and the responses were recorded using a TCD detector. We assumed that the stoichiometric ratio of number of adsorbed CO molecules and number of accessible Pd surface atoms is one. Pd particle size was determined using TEM (Tecnai F30), measuring at least 300 Pd particles at ten different spots in the catalyst. The metal dispersion using TEM average particle size was calculated using the method proposed elsewhere (see **Equations S1-3**).

### 2.4 Ultrasonic bath and CIB characterization

A BANDELIN Sonorex Digitec ultrasonic bath with a frequency of 35 kHz and content capacity of approximately 7 L was characterized by sonicating a solution of 1x10^-3^ mol/L of Luminol (Sigma-Aldrich) prepared in deionised water and pH 12 adjusted by adding Na_2_CO_3_ (Sigma-Aldrich) [29]. In this way, we identified the sonochemical active regions inside the ultrasonic bath, where hydroxyl radicals react with Luminol, and luminescence with an intensity proportional to the number of radicals is produced [30], [31].

**Scheme 1:**
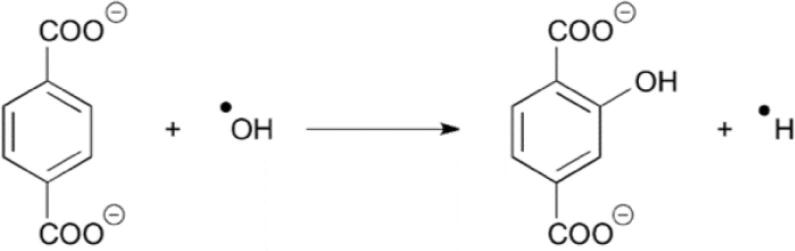
Oxidation of terephthalic acid (TA) to 2-hydroxyterephthalic acid (HTA) in the presence of hydroxyl radicals in basic media [36], [37].

The conversion of terephthalic acid (TA) to 2-hydroxyterephthalic acid (HTA) was taken as a quantitative measure of the concentration of ^.^OH radicals formed by the ultrasound induced cavitation (see Scheme 1). The terephthalic acid solution used as dosimeter was prepared by mixing 0.3323 g of terephthalic acid (2.0 mM, Sigma-Aldrich), 0.2000 g of NaOH (5.0 mM, Sigma-Aldrich) and phosphate buffer (pH 7.4), prepared from 0.5988 g of KH_2_PO_4_ (4.4 mM , Riedel-de Haen) and 0.9937 g of Na_2_HPO_4_, (7.0 mM, Riedel-de Haen). The resulting solution was then made up to 1 L with water [32]. In a typical experiment, 150 mL of terephthalic acid solution were placed in a reaction bag. Two types of bags were employed in these experiments, including; Cavitation Intensifying Bags (CIB) (BuBclean) [33] containing surface pits and conventional bags with smooth inner surface and identical dimensions as the CIB. The CIB are made of poly-propylene polymer. This material is sufficiently robust to withstand the sonication environment without noticeable degradation [21]. We designed and built 3D printed bag holders to avoid fluctuations in the positioning of the bags during experiments (see Fig. S1). For this purpose, we employed the Ultimaker printer system using Nylone (PA2200) polymer [34]. An active temperature control system was operated during the sonication process by pumping water with an external water bath (Clifton 28 L), cooler (Clifton 700 W) and pump (Masterex Console Drive and attachment Masterex L/S Easy-load II). These measures were taken to minimize the experimental errors associated to degassing of the liquid inside the bath due to increased temperature. To test the sonocatalytic degradation of TA we conducted experiments in the presence of Al_2_O_3_ and Pd/Al_2_O_3_. Like in the sonochemical experiments, in the sonocatalytic tests 300 mL of buffered TA solution were loaded in the CIB in combination with a weighted mass of catalyst (10–90 mg). For each sonication time (e.g. 5–45 min) we conducted four experiments in order to determine the average and standard deviation of the reactants and products concentrations using a spectrofluorometer (HORIBA Scientific FluoroMax) with an excitation wavelength at 310 nm and emission wavelength of 425 nm (Fig. S3) [35].

## 3 Results

### 3.1 Characterization

**Fig. 2:**
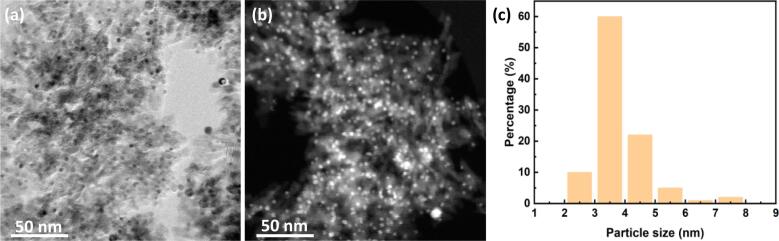
Electron microscopy characterization of the Pd/Al_2_O_3_ via (a) bright and (b) dark field HR-Transmission Electron Microscopy, and (c) particle size distribution of Pd clusters.

The Pd loading of the prepared catalysts was 4.1 wt%, which is close to the targeted values of 5 wt%. The metal dispersion for this catalyst sample was 28%. The report of micro-XRF, metal dispersion by CO-chemisorption and particle size using HRTEM is provided in the Appendix C. Further characterization by HR-TEM using bright and dark field electron microscopy revealed that Pd cluster are well dispersed on the Al_2_O_3_ surface with an average particle size of 3.9 ± 0.9 nm (see Fig. 2). As shown in Fig. S4 and Table S1, the dispersion measurements of Pd obtained by CO-Chemisorption (40.6%) were close to those resulting from the analysis of particle size distribution obtained by HRTEM [38] (30%), which is in line with previous results recently reported on Pd/Al_2_O_3_ catalysts [28], [39]. During the catalyst synthesis the Pd/Al_2_O_3_ catalyst is reduced in hydrogen. However, upon exposure to air at room temperature the Pd surface can be re-oxidized [40], [41], [42], [43]. In fact, investigations of the oxidation state of Pd supported on alumina catalyst, with equivalent metal cluster size, revealed that the majority of the Pd is in the metallic state upon exposure to air after the reduction treatment, with a surface atomic ratio of Pd^0^:Pd^2+^ of 2.3 (see Fig. S7 and Table S3).

### 3.2 Sonochemical degradation of TA

The results from the ultrasonic bath characterization indicate that during sonication, an uneven distribution of hydroxyl radicals is observed as sonochemiluminescence is primarily occurring in the center of the bath (see Fig. S2). Notably, under stirring the luminescence areas were redistributed, which leads to more homogeneous conditions desired in the experiments with CIB, particularly in keeping the temperature and any degassing effect to a minimum. The determined positions of larger luminol intensity were marked to place the CIBs and the normal bags throughout all remaining experiments.

**Fig. 3:**
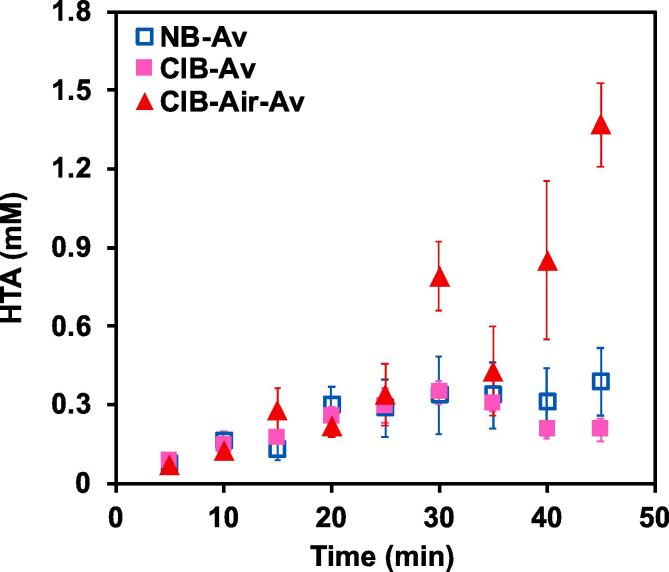
Concentration of 2-hydroxyterephthalic acid (HTA) as a function of time during (a) sonication of TA solutions inside of normal bags (NB-Av) and cavitation intensifying bags in the absence of air flow (CIB-Av), and in the presence of air bubbling at 25 °C in the CIB (CIB-Air-Av). The initial concentration of terephthalic acid was 2.0 mM, while NaOH KH_2_PO_4_ and Na_2_HPO_4_ concentrations were 5.0 mM, 4.4 mM, and 7.0 mM, respectively.

The activity of CIB and normal bags (NB) during the oxidation of the water-soluble organic molecules was studied first in the absence of catalyst employing terephthalic acid (TA) as a dosimeter. This prototypic reaction allows effective scavenging of the hydroxyl radicals produced during the ionization of water molecules near the interface of the cavitating bubbles [25]. As it is shown in Fig. 3a, the initial rate of HTA formation seems to be similar during the first twenty minutes with values of 0.67 and 0.65 μM/min for the NB and CIB systems, respectively. A detailed analysis of the standard deviations of the average HTA concentration obtained after 25 min of reaction showed that on the normal bags a wider spread of concentrations were observed when compared to the CIB (see Table S2). This is in agreement with previous reports indicating that CIBs facilitate better control and reproducibility of the sonication process in comparison with the conventional bags i.e. without pits [36]. The observed concentration of HTA in the solution as a function of time during sonochemical oxidation in the CIBs also resembles the one obtained in previous reports [14], [21], [25], [36]. We then followed previous estimations on the number of pits present in each CIB, the corresponding number of bubbles and radicals produced per pit [44]. This gives approximate values of the radicals produced per CIB. The calculated values compared with the determined radical production are shown in Fig. S5a. Notably, the calculated number of hydroxyl radicals closely resembles that obtained from experiments as it can be noticed by the nearly linear correlation obtained in the Pareto plot (see Fig. S5b) in the first 30 min of sonication. However, at long sonication times (>30 min) the rate of HTA formation in the CIB and NB decreased.

The asymptotic behavior of HTA concentration after 30 min of reaction, on both types of bags, could be associated to undesired degradation of HTA to oxidation products masking the real production rates of ^.^OH radicals formation. To unravel the underlying mechanism controlling the sonication performance, we conducted experiments of HTA stability and TA oxidation with continuous gas bubbling during sonochemical reaction. As shown in Fig. S5, the initial HTA concentration (0.75, 1.00, and 2000 μM) did not change even after prolonged periods of acoustic radiation (~50 min), indicating that the drop in HTA formation observed during experimentation in the CIB and normal bags was not caused by sequential oxidation. Alternatively, this behavior could be attributed to a decrease in the concentration of gas dissolved in the solution and removal of the bubbles trapped inside the surface pits of the CIB, leading to a reduction in the number of cavitating bubbles and, subsequently, a slower TA oxidation rate. This reduction originates from the action of the ultrasound, which accelerates degassing of the liquid and undesired temperature increase in the bath. While the solubility of gasses in liquids decreases with increasing temperature, the measured temperature increase was not larger than 3–3.5 °C after 50 min. Such minor change in the temperature will not cause a drastic change in the solubility. Instead, we attributed the activity drop to ultrasound-induced degasification [12], [37].

To test our hypothesis, we conducted experiments in the CIB with continuous bubbling of air (~100 mL/min) during oxidation of TA (see Fig. 3**b**). Initially, the addition of air in the system did not significantly affect the rate of TA oxidation to HTA (first 25 min). However, at longer sonication times, the rate of HTA formation drastically increased three-fold, for the system with continuous bubbling of air. This indicates that sparging air during the sonochemical oxidation of organic molecules can improve the performance of our system by replenishing the dissolved and trapped gas inside the CIB.

**Fig. 4:**
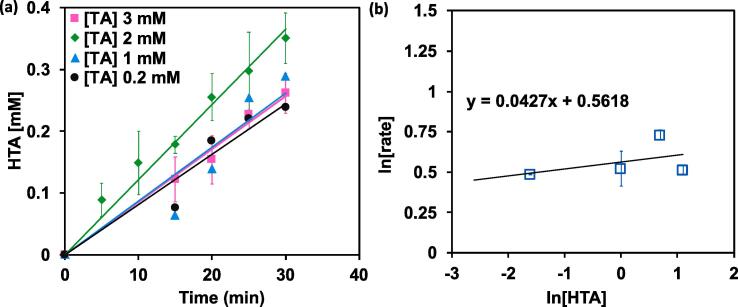
Evolution of the (a) concentration of HTA as function of time during sonication in CIB at different initial concentrations of TA (0.2, 1, 2, and 3 mM) and (b) log-log analysis of the initial rates showing the low reaction order of TA oxidation in CIB (indicated by the slope) attributed to “saturation kinetics” where the HTA formation quickly occurs as hydroxyl radical are generated during the cavitation of the bubbles.

The interplay of the oxidation kinetics in the sonication with the initial concentration of TA was studied in the absence of catalyst to determine the reaction orders. For this purpose, we conducted a series of experiments, with quadruplet repetitions, varying the initial concentration of the TA in the CIB reaction system. The initial rates for each experiment were calculated by extrapolating to time zero the rate of HTA formation (Fig. 4a). Notably, this analysis revealed that the rate of HTA formation was independent of the TA initial concentrations with a reaction order of ~0.04 (Fig. 4b). The low reaction order in TA could be attributed to “saturation kinetics” where the HTA formation quickly occurs as hydroxyl radical are generated during the cavitation of the bubbles.

### 3.3 Sonocatalytic degradation of TA

**Fig. 5:**
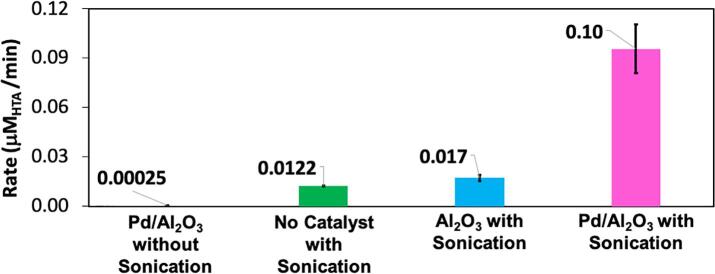
Initial reaction rates of HTA formation for Al_2_O_3_ metal oxide with sonication, Pd/Al_2_O_3_ catalyst without sonication, no catalyst without sonication, and Pd/Al_2_O_3_ catalyst with sonication after 50 min of reaction. The catalyst mass was 50mg using TA initial concentration of 2 mM in 300 mL of DI H_2_O in a CIB.

Since the study of catalysts in the CIB has not been explored before, we evaluated the performance of the support (Al_2_O_3_) and catalyst (Pd/Al_2_O_3_) for the oxidation of TA under different reaction conditions, by assessing the initial rates of HTA formation (see Fig. S6a-d). In the silent conditions, the Pd/Al_2_O_3_ catalyst generated a negligible amount of HTA leading to reaction rates of ~0.0003 μM_HTA_/min, which are significantly lower than those observed during sonication in the absence of catalyst (0.01 μM_HTA_/min), see Fig. 5. When using the support (Al_2_O_3_) under sonication, the formation of HTA reached values of ~0.017 μM_HTA_/min, which is statistically indistinguishable from the base case in the absence of catalyst (see Table S2). This confirms that Al_2_O_3_ is inert towards the activation of TA oxidation chemistry. Notably, combining Pd/Al_2_O_3_ catalyst with sonication increased the reaction rate by an order of magnitude, reaching values of ~0.1 μM_HTA_/min. While previous studies found similar enhancements in the catalytic activity when combining catalysts and sonication, our study indicates that using the CIBs enhance the performance with well-controlled and reproducible cavitation CIBs where higher energy efficiencies can be achieved [21]. This enhancement has been previously attributed to a combination of improved mass transport and the creation of thermal gradients near the cavitating bubbles (~2 nm), where the combined action of the catalyst to activate the substrates and the oxidation radicals leads to exponential increase in the reaction rates [45], [46], [47], [48].

Detailed inspection of the reaction kinetics in the presence of Pd/Al_2_O_3_ revealed that HTA formation initially was negligible, achieving only HTA concentrations of ~0.3 mM after 20 min reaction with zero derivative at zero time. As time evolved the slope increased, reaching concentrations as high as 6 mM of HTA after 40 min, indicating an induction of activity. Extending further the reaction time, however, did not lead to additional HTA formation. While the asymptotic behavior at longer reaction times could be associated to the depletion of dissolved gas and removal of trapped gas bubbles in the CIB pits [44], as we observed in the non-catalytic experiments (see Fig. 3), the exact nature of the initial induction period remains unknown to us.

One could argue that this behavior resembles that often observed on heterogenized organometallic catalysts in which the metal center lixiviates from the catalyst surface forming an “active” homogeneous catalyst [49]. In our case, however, the mild reaction conditions employed (e.g. low sonication power , room temperature, and buffered pH) do not explain leaching of Pd-clusters from the Al_2_O_3_. In the past, we have demonstrated that Pd-Al_2_O_3_ catalysts are hydrothermally stable materials capable of withstanding the harsh reaction conditions employed in biomass hydrotreating upgrading [50], [51], [52]. Furthermore, the results of the stability study of Pd supported on Al_2_O_3_ indicated that even after 4 h of continuous exposure to sonic radiation the catalyst chemical composition did not significantly change (see Table S4). Thus, metal-dissolution into the bulk of the solution seems unlikely. A more plausible alternative is the formation of hydroperoxides on the palladium surface, which slowly form on the catalyst via a complex free radical mechanism [53], [54]. Upon decomposition, these hydroperoxides intermediates generate free radicals that further attack other organic species, accelerating the overall reaction. In previous studies we observed similar trends during the catalytic oxidation of tetralin to 1-tetralone and 1-tetralol on Pd and Cu catalysts supported on nanohybrids comprising single walled carbon nanotubes anchored to silicon dioxide [55]. In that case, induction periods of 18 and 24 h were obtained for Cu and Pd, respectively, in which tetralin 1-hydroperoxide readily decomposed to yield 1-tetralone and 1-tetralol, the two main products experimentally observed.

**Fig. 6:**
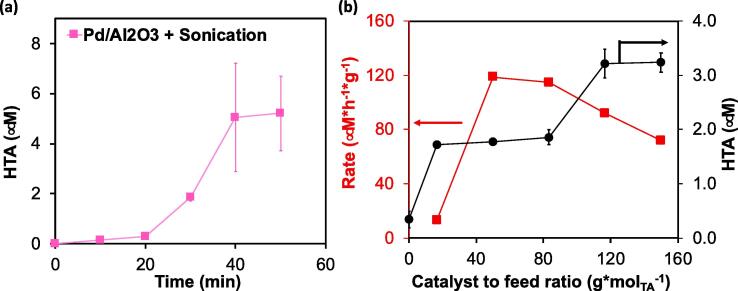
Evolution of HTA concentration with sonication time when using 50 mg of Pd/Al_2_O_3_ (a) and reaction rate per mass of catalyst (red) and HTA final concentration (black) as a function of the catalyst to feed ratio obtained during the oxidation TA using Pd/Al_2_O_3_ after 0.5 h of reaction (b). The experiments were conducted in a CIB of 0.3 L with an initial concentration of terephthalic acid of 2.0 mM in combination with NaOH KH_2_PO_4_ and Na_2_HPO_4_ concentrations of 5.0 mM, 4.4 mM, and 7.0 mM, respectively. (For interpretation of the references to colour in this figure legend, the reader is referred to the web version of this article.)

To study the interplay between the sonocatalytic activity observed and the available catalyst we conducted a set of experiments varying the catalyst to feed ratio in the CIB system (see Fig. 6b). The results indicate that initially the concentration of HTA after 0.5 h of reaction increased as a function of the catalyst mass. At catalysts to feed ratios above 120 g_cat_*mol_TA_^-1^ the concentration of HTA plateaued at values of c.a. 3.2 μM (black circles). Non-linear correlation between the formation of product with the available catalyst would be indicative of mass and/or heat transport limitations in the system. This is observed in the trends of mass-normalized reaction rates (red squares) where the rate of reaction increased until the catalyst to feed ratio reached 80 g*mol^−1^. After this value, the reaction rate decreased. It is not surprising that in this system quickly after 30 min of reaction, when the rate exponentially increased upon offsetting the induction period, the reaction runs into diffusional limitations, which lead to catalyst underutilization.

#### 3.3.1 Process Intensification Factor analysis

The advantages of using the CIB when compared with NB has already been demonstrated with respect to an improved energy efficiency [56]. We now use the same comparison method in the context of adding a catalyst, by using the Intensification Factor (IF) which can be defined as:

$$\mathrm{IF}=\prod_{i=1}^{n}{\left(\frac{F_{b}}{F_{a}}\right)}^{d}$$

where F is a specific factor such as the number of radicals produced, cost, the yield of a given reaction, or the residence time through a reactor to allow a reaction to occur. For a given factor F, we have as input data its initial value F_b_, and the value of the same factor after the modifications is F_a_. The meaning of the absolute value of the exponent *d* is determined depending on the intensification target. The value of “n” corresponds to the number of factors or aspects available for comparison. As we are interested in the increased oxidative power of ultrasound, a decrease in TA concentration is seen as “desirable”, which corresponds to an exponent d = -1 for the increase in HTA. With the limited information we have for this system, we included reaction rate and cost due to the metal in the catalyst, and combined it (n = 3) in a global IF_total_. We builtt Table 1 to compare CIBs with and without catalyst. As it can be observed, IF_total_ has a large positive value (~42), which indicates that the CIB in combination with our catalyst leads to an improvement in the oxidation and process efficiency for the oxidation of TA.

**Table 1:** Intensification Factor calculations for CIBs with and without catalyst.

Factor	CIB	CIB + Catalyst	d	Fraction	IF
HTA concentration [µM L^-1^] in 30 min	0.35	1.85	−1	(0.35/1.85)^-1^	5.29
Reaction rate [µM min^−1^] (assuming linear behavior)	0.012	0.096	−1	(0.012/0.096)^-1^	8
Cost due to metal in catalyst [Euro/kg] in % ±	100	260	1	100/260	0.4
				IF_Total_	=42.32

± Assuming the price of catalyst is controlled by the price of the metal, taken as 61 $/g for Pd, with 4.1% Pd loading, and 50 mg of catalyst used in each experiment, the resulting price of producing an OH^.^ radical (5.25 µM) was 0.12 $/g. – 160 Euro/kg (typical Pd based catalyst) [57].

As discussed elsewhere [56], this is a method that helps to get a simplified overview of different alternatives, particularly when data is limited. With subsequent studies, the number of “factors” can be increased and a new IF_total_ must be calculated. We assumed a linear behavior of the factors considered, which can be true in some cases, e.g. due to improved mass transfer which is a first order phenomenon. In real life scenarios, any efforts of scaling up the CIBs must tackle more practical difficulties, such as those imposed by the cost of using catalysts. In this particular, academic example, the cost is less of a problem dwarfed by the improved outcome in the other two factors.

## 4 Conclusions

The novel reactor concept based on Cavitation Intensifying Bags has been characterized with Pd/Al_2_O_3_ catalysts, showing improved performance as an Advanced Oxidation Process. We have found that increasing the catalyst ratio, initially increases the rate of conversion; however, at higher ratios, its effect vanishes, due to insufficient heat available reaching the active catalyst sites. Moreover, we found that sparging air during the sonochemical oxidation of TA improves the performance by replenishing the dissolved and trapped gas inside the CIB. Our preliminary Process Intensification Factor analysis indicates that sonicating in the CIB with catalysts is overall beneficial, despite the high catalyst cost. One possible avenue to explore further scale-up strategies is to build a flow reactor based on this batch system.

## CRediT authorship contribution statement

**Maria Pappaterra:** Data curation, Investigation, Visualization, Writing - original draft. **Pengyu Xu:** Data curation, Investigation, Visualization. **Walter van der Meer:** Conceptualization, Funding acquisition, Methodology, Validation, Writing - review & editing. **Jimmy A. Faria:** Conceptualization, Investigation, Methodology, Supervision, Visualization, Writing - original draft, Writing - review & editing. **David Fernandez Rivas:** Conceptualization, Funding acquisition, Investigation, Methodology, Project administration, Resources, Supervision, Visualization, Writing - original draft, Writing - review & editing.

## Declaration of Competing Interest

The authors declare the following financial interests/personal relationships which may be considered as potential competing interests: D.F.R. is a founder and a scientific advisor of BuBclean, a Dutch start-up company that commercializes the BuBble Bags (Cavitation Intensifying Bags – CIB) and operates in the ultrasonic cleaning solutions market. W.vd.M, besides being professor at the University of Twente, is CEO of OASEN. No conflict of interest has been identified in both cases.
