# Supplementary material for: Cavitation intensifying bags improve ultrasonic advanced oxidation with Pd/Al2O3 catalyst
Source: Ultrason Sonochem. 2020 Sep 7;70:105324. doi: 10.1016/j.ultsonch.2020.105324 (PMC7786540; doi:10.1016/j.ultsonch.2020.105324)
Supplement: Supplementary data 1 [file mmc1.docx]

**Supporting Online Information**

1. **US bath and BB characterization**

**Figure S1:** Digital design of the reaction bags holder (a) and 3D printed system for positioning of the cavitation bags employed in the sono- chemical and catalytic degradation experiments conducted in the ultrasonic bath.

**Figure S2:** Top view of a) stagnant conditions and b) continuously stirred Luminol solution inside the ultrasonic bath. The CIB were positioned above the most active areas matching with higher sonochemiluminescence intensity (c).

1. **Calibration of HTA**

**Figure S3:** Calibration curve for 2-Hydroxyterephthalic acid (HTA) in aqueous solution obtained using a spectrofluorometer (HORIBA Scientific FluoroMax) with an excitation wavelength at 310 nm and emission wavelength of 425 nm.

1. **CO-Chemisorption on Pd/Al2O3**


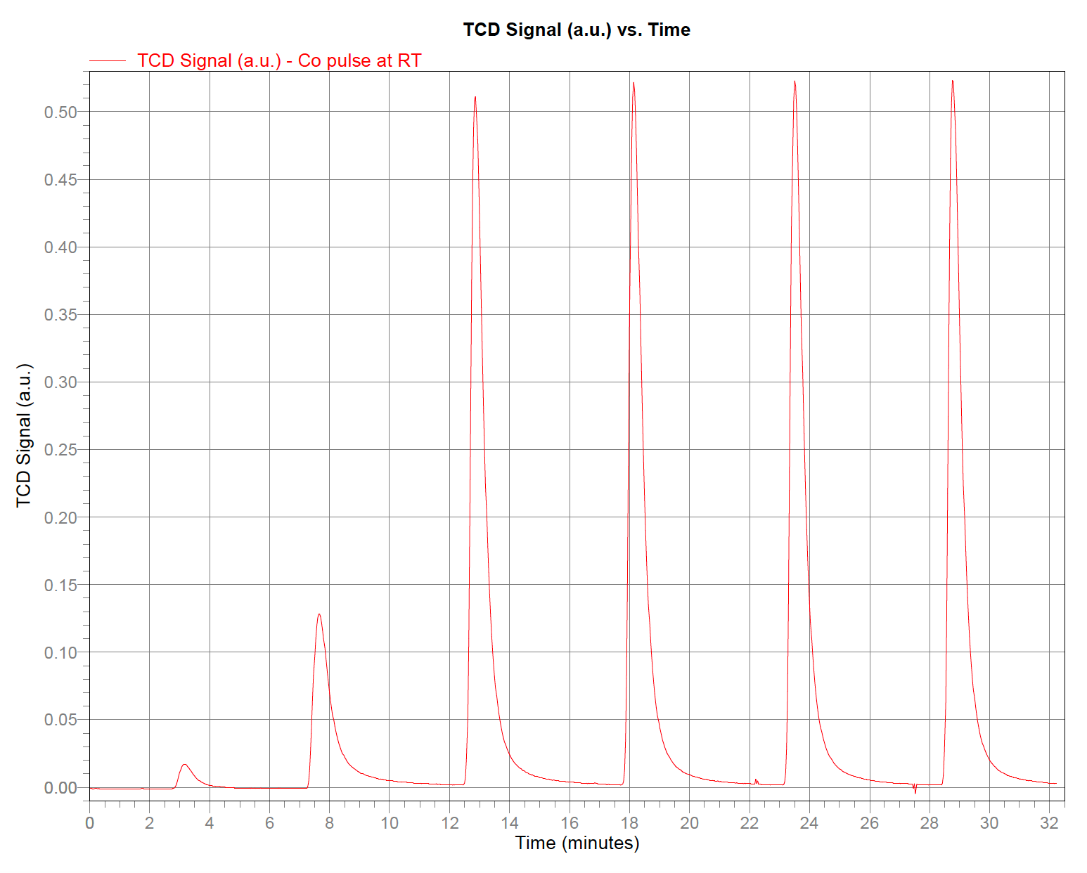


**Figure S4:** Carbon monoxide chemisorption on Pd/Al_2_O_3_ (0.0529 g) after purge with He for 20 minutes at room temperature and reduction in H_2_ for 1 hour. Followed by flushing with He for 30 minutes.

**Table S1:** Pulse chemisorption analysis summary.


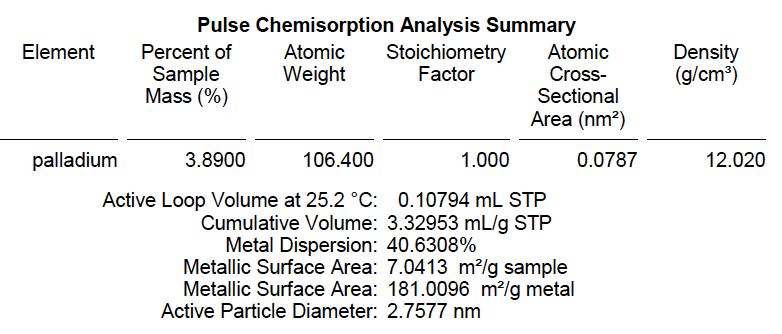


1. **Determination of the metal particle size from HR-TEM characterization of the average particle size**

***Assumptions:***

*The stoichiometry factor for CO on Pd is 1.*

*The Pd is hemisphere, and the size is uniform.*

***Calculation is shown below***[58]*.*

*Vchem (mol*g^-1^) : chemisorption volume*

*MW (g*mol^-1^) : supported metal atomic weight*

*σ_m_ (nm^2^) : supported metal cross section area*

*SF : stoichiometry factor*

*c (wt%) : supported metal weight percent*

*ρ (g*cm^-3^) : supported metal density*

*r (m) : supported metal radius*

*S_m_ (nm) : supported metal diameter*

*A_m_ (m^2^*g) : supported metal surface area per catalyst*

*Dm (%) : supported metal dispersion*

***Information about Pd/γ-Al_2_O_3_***

*MW=106.42 g*mol^-1^*

*σ_m_ = 0.0787 nm^2^*

*SF (CO) = 1*

*c = 4.1 wt%*

*ρ = 12.023 g*cm^-3^*

*Mean particle diameter,*

$S_{m}=2*r*{10}^{9}=\frac{6*c}{A_{m}*100*\rho*{10}^{6}}*{10}^{9}=\frac{60c}{A_{m}*\rho}$ ***Eq. S1***

*Metal surface area (per catalyst),*

$A_{m}=V_{chem}*6.02*{10}^{23}*SF*\sigma_{m}*{10}^{-18}$ ***Eq. S2***

*Metal dispersion,*

$Dm= \frac{Chemisorption site}{Supported metal atomicity}*100=\frac{V_{chem}*SF*MW}{c/100}*100$ ***Eq. S3***

*Sm = 3.85 nm.*

*A_m_ = 5.303 m^2^*g*

*Dm = 29 %*

*The results are equivalent to the co-chemisorption in the error margin.*

1. *Kinetic Study of TA oxidation*

**

***Figure S5:*** *Number of hydroxyl radicals (*OH) inside the CIB reactor (a) as function of time obtained from experimental and theoretical estimations and (b) the Pareto plot of the calculated and experimental *OH radicals.*

**Figure S6:** Evolution of HTA concentration as a function of time during reaction on CIB bags at different operating conditions for Al_2_O_3_ metal oxide with sonication, Pd/Al_2_O_3_ catalyst without sonication, no catalyst without sonication, and Pd/Al_2_O_3_ catalyst with sonication. The catalyst mass was 50 mg using TA initial concentration was 2 mM in 300 ml of DI H_2_O in a CIB.

**Table S2:** Average 2-Hydroxyterephthalic acid (HTA) concentration as a function of time on normal (NB) and Cavitation Intensifying Bags (CIB).

| **Time (min)** | **Cocentration (μM)** | |
| --- | --- | --- |
|  | **NB** | **CIB** |
| 5 | 0.08 ± 0.01 | 0.09 ± 0.03 |
| 10 | 0.17 ± 0.02 | 0.15 ± 0.05 |
| 15 | 0.13 ± 0.04 | 0.18 ± 0.01 |
| 20 | 0.30 ± 0.07 | 0.25 ± 0.04 |
| 25 | 0.3 ± 0.11 | 0.30 ± 0.06 |
| 30 | 0.3 ± 0.15 | 0.35 ± 0.04 |
| 35 | 0.3 ± 0.13 | 0.30 ± 0.03 |
| 40 | 0.3 ± 0.13 | 0.21 ± 0.03 |
| 45 | 0.4 ± 0.13 | 0.21 ± 0.04 |
| 50 | 0.64 ± 0.05 | 0.37 ± 0.06 |

1. HTA degradation study

**Figure S6:** HTA degradation as a function of time during sonication in the CIB at different initial concentration of HTA (0.75, 1.00, and 2000 μM) with NaOH KH_2_PO_4_ and Na_2_HPO_4_ concentrations of 5.0 mM, 4.4 mM, and 7.0 mM, respectively.

1. Additional characterization of Pd catalyst

We synthesized a second sample of catalyst containing 1 wt. % Pd/Al_2_O_3_ , using the same method as in 4.1 % Pd/Al_2_O_3_, for the characterization of the oxidation state of the metal. While the 1 wt. % Pd/Al_2_O_3_ sample had a smaller content of palladium the metal dispersion and cluster size obtained from CO-chemisorption resembled that of the 4.1 wt. % Pd/Al_2_O_3_ catalyst with values of 38 % and 2.8 nm, respectively (see **Table S3**). Since the metal cluster size and support were the same as that of the 4.1 wt. % Pd/Al_2_O_3_, we could employ this reference sample to investigate the oxidation state of the catalyst before the reaction.

The X-ray photoelectron spectroscopy (XPS) was conducted in an Omicron Nanotechnology GmbH (Oxford Instruments) surface analysis system with a photon energy of 1486.7 eV (Al Kα X-ray source) with a scanning step size of 0.1 eV and a pass energy of 20 eV. Due to the poor electrical conductivity of sample surface, it is necessary to neutralize charge on the sample with an electron spray. The spectra were corrected using the binding energy of C 1s peak as a reference. The peak deconvolution was conducted with Casa XPS.

As it is shown in Figure S7, the characteristic peaks for the Pd(3d^5/2^) and Pd(3d^3/2^) spin orbit doublet at approximately 334.5 and 340.6 eV were observed in the 1 wt. % Pd/Al_2_O_3_. However, the XPS peaks around 336.3 (Pd^2+^ 3d5/2) and 344.8 eV (Pd^2+^ 3d3/2) are observed indicating that part of the Pd was oxidized in the atmosphere, which is in good agreement with the literature [59]. The analysis of the deconvoluted spectra (CasaXPS) indicated that the ratio of Pd zero to 2+ was ~ 2.3 (see Table S3). This indicates that the fraction of oxidized palladium was significantly smaller than that of metallic Pd.

**
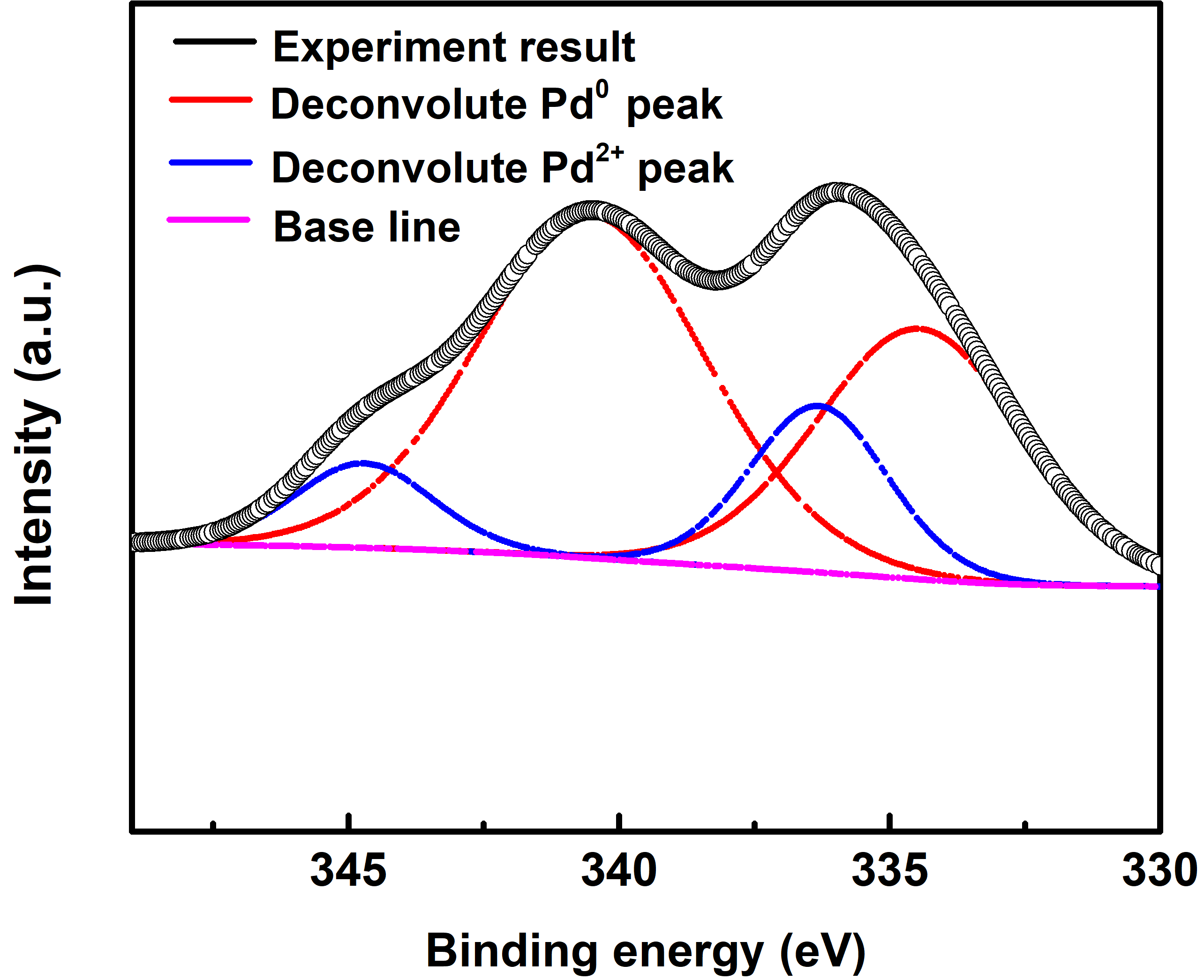
**

**Figure S7:** XPS spectra of fresh Pd/γ-Al_2_O_3_ catalyst kept under ambient atmosphere (1 wt. % Pd).

**Table S3.** Pd/Al_2_O_3_ Characterization.

| Catalyst | Pd/γ-Al_2_O_3_ |
| --- | --- |
| Catalyst specific surface area, m^2^/g | 195 |
| Metal loading, wt% | 0.9 |
| Metal dispersion, % | 38 |
| Mean metal particle size, nm | 2.8 |
| Pd^0^/Pd^2+^ | 2.3 |

1. Catalyst stability during sonication

To determine the extent of metal leaching in aqueous environments during sonic radiation we conducted a stability experiment in which a catalyst containing 1 wt. % of Pd/Al_2_O_3_ was exposed to prolonged sonication treatment. In a typical experiment 0.20 g of catalyst with a particle size blow 45 μm was dispersed on 40 g of milliQ water in a centrifuge tube. The dispersion was sonicated for 4 hours at room temperature using a sonication bath. Then catalyst was separated in the centrifuge at 5000 rpm for 5 min. The separated catalyst was dried in a vacuum oven at 35 ^o^C for 24 hours. The resulting powder was sent to μ-XRF analysis. As it can be seen in **Table S4**, the chemical composition of the catalyst after extended sonication treatment remained constant. This indicates that the sonication processes did not significantly change the metal loading of the catalyst. The major differences were observed on the concentration of the S, Cl, and K. However, these elements were present in trace quantities. These elements are part of the aluminum oxide powder employed as support.

**Table S4:** Chemical composition of the 1 wt. % of Pd/Al_2_O_3_ determined by μ-XRF before and after sonication treatment in aqueous environment.

|  | Before | After |
| --- | --- | --- |
| Al_2_O_3_ | 98.10% | 97.90% |
| Pd | 0.77% | 0.88% |
| P | 0.43% | 0.39% |
| Ti | 0.24% | 0.25% |
| Ca | 0.17% | 0.12% |
| Cl | 0.12% | 0 |
| S | 797 ppm | 0 |
| K | 0 | 258 ppm |
| Fe | 214 ppm | 254 ppm |
| Cu | 179 ppm | 199 ppm |
| Zr | 104 ppm | 72.6 ppm |
| Zn | 99.5 ppm | 100 ppm |

**References**

[1] K. Nakai, K. Nakamura, Pulse chemisorption measurement Metal dispersion measurement, 6, Adsorpt. J. Int. Adsorpt. Soc. (2003) 1–6.

[2] H.J. Jeon, Y.M. Chung, Hydrogen production from formic acid dehydrogenation over Pd/C catalysts: Effect of metal and support properties on the catalytic performance, Appl. Catal. B Environ. 210 (2017) 212–222. https://doi.org/10.1016/j.apcatb.2017.03.070.
